# Comparison of two-drug combinations, amikacin/tigecycline/imipenem and amikacin/tigecycline/clarithromycin against *Mycobacteroides abscessus* subsp. *abscessus* using the in vitro time-kill assay

**DOI:** 10.1038/s41429-020-00392-0

**Published:** 2021-01-08

**Authors:** Elena Portell-Buj, Queralt Bonet-Rossinyol, Alexandre López-Gavín, Angely Roman, Mariana Fernández-Pittol, Griselda Tudó, Julian Gonzalez-Martin

**Affiliations:** 1 Departament de Fonaments Clínics, Facultat de Medicina i Ciències de la Salut, Universitat de Barcelona, Servei de Microbiologia, CDB, Hospital Clínic de Barcelona-ISGlobal, c/ Villarroel 170, 08036 Barcelona, Spain; 2 Servei de Microbiologia, CDB, Hospital Clínic de Barcelona-ISGlobal, Departament de Fonaments Clínics, Facultat de Medicina i Ciències de la Salut, Universitat de Barcelona, c/ Villarroel 170, 08036 Barcelona, Spain; 3 Laboratory of Molecular Microbiology, Biology Department, University of Girona, Girona, Spain

**Keywords:** Bacterial infection, Respiratory tract diseases

## Abstract

Nontuberculous mycobacteria include 198 mycobacterial species. Among these, *Mycobacteroides abscessus* is a rapidly growing mycobacteria that causes lung and skin infections. *M. abscessus* lung infections are difficult to treat due to the high levels of resistance to several classes of antibiotics. The current treatment is based on combining at least two or three antibiotics. However, treatment outcomes remain very poor. The objective was to compare the in vitro activity of amikacin, tigecycline, imipenem, and clarithromycin, alone and in two different three-drug combinations (amikacin/tigecycline/imipenem and amikacin/tigecycline/clarithromycin) against seven *M. abscessus* subsp. *abscessus* clinical isolates using the time-kill assay. The two combinations showed greater activity than the antibiotics tested individually. Even though both combinations showed similar activity as well as no antagonistic activity, the combination including imipenem could not be an alternative treatment against *M. abscessus* subsp. *abscessus* lung infections caused by clarithromycin susceptible isolates. However, this combination could be considered against clarithromycin resistant isolates. Future studies are necessary to confirm this hypothesis.

## Introduction

The nontuberculous mycobacteria (NTM) family includes 198 species, the majority of which do not cause disease in humans [1]. NTM are naturally found in the environment, from soil to water systems [2]. Recently, mycobacterial species have been divided into five new genera: *Mycobacterium, Mycolicibacter, Mycolicibacterium, Mycolicibacillus, and Mycobacteroides* [3]. Over the last years, infections caused by NTM have significantly increased [4]. Among these NTM, *Mycobacteroides abscessus* is a rapidly growing mycobacteria (RGM) that causes lung and skin infections which are difficult to treat due to its resistance to most classes of antibiotics [2, 4]. This RGM is classified into three subspecies: *M. abscessus* subsp. *abscessus*, *M. abscessus* subsp. *massiliense* and *M. abscessus* subsp. *bolletii* [2, 3, 5]. The identification to subspecies level is important since their resistance profiles to macrolides are different. Macrolides, and specifically clarithromycin are among the most active antibiotics against *M. abscessus*. The resistance to macrolides can be either constitutive (*rrl* gene) or inducible (*erm*41 gene). The most frequent is the inducible resistance, due to the presence of a functional *erm*41 gene that codes for a methylase. Accordingly, *M. abscessus* subsp. *massiliense* is usually susceptible to macrolides since the *erm*41 gene is truncated and not functional. *M. abscessus* subsp. *bolletii* is innately resistant to macrolides. Most of *M. abscessus* subsp. *abscessus* also exhibit inducible macrolide resistance, but some isolates remain susceptible due to a non-functional *erm*41 gene. Although there are geographical differences, the most commonly isolated is *M. abscessus* subsp. *abscessus* [4, 6].

*M. abscessus* lung infections are particularly common in patients with underlying respiratory conditions, such as cystic fibrosis and bronchiectasis [5]. These infections require treatment, but management is difficult due to high levels of natural and acquired resistance to frequently used antibiotics. Moreover, the presence of mucus and other secretions at the site of infection makes the entry of antibiotics difficult. Depending on the macrolide resistance profile, the current guidelines of the British Thoracic Society recommend the administration of different intravenous (IV) and oral antibiotics (e.g., IV amikacin, IV tigecycline, IV imipenem, and oral clarithromycin) for 1 month during the initial phase followed by oral and inhaled antibiotics (e.g., oral clofazimine, oral moxifloxacin and nebulized amikacin) for a minimum of 12 months during the continuation phase. The use of macrolides is recommended even in the presence of inducible resistance, but not when constitutive resistance associated to the *rrl* gene is found [6, 7]. On the other side and despite its IV administration, tigecycline has better activity than former drugs used in combination with amikacin, such as doxycycline, tetracycline and minocycline [8]. Nevertheless, side effects are common and treatment outcomes are very often poor [2]. Hence, it is of great importance to develop new antibiotics and antibiotic combinations.

The treatment regimens available for *M. abscessus* lung infections consist of the administration of different antibiotics in combination. Therefore, it is necessary to determine the efficacy of these combinations as well as of that of the antibiotics alone. The time-kill assay establishes the in vitro pharmacodynamics of antibiotics by detecting the rate at which an antibiotic concentration kills bacteria along time [9].The main goal was to compare the in vitro activity of four different antibiotics, amikacin, tigecycline, imipenem, and clarithromycin alone and in two different three-drug combinations (amikacin/tigecycline/imipenem and amikacin/tigecycline/clarithromycin) against *M. abscessus* subsp *abscessus* clinical isolates using the in vitro time-kill assay.

## Material and methods

### Mycobacteroides abscessus subsp. abscessus clinical isolates

Seven clinical isolates of *M. abscessus* subsp. *abscessus* were selected from a strain collection of the Microbiology Department of the Hospital Clinic of Barcelona (Spain). They were previously identified at subspecies level using GenoType^®^ NTM-DR (Hain Lifescience GmbH, Nehren, Germany). In addition, Sensititre™ AST RAPMYCO plates (Thermo Fisher Scientific, MA, USA) were used in accordance to manufacturers recommendations for antibiotic susceptibility testing, incubating the plates for 3 days, except for clarithromycin whose incubation was prolonged for up to 7 and 14 days to detect the presence of inducible resistance. The seven isolates selected had the following range of minimum inhibitory concentrations (MICs) to the antibiotics tested: 4–16 µg ml^−1^ for amikacin, 0.125–0.5 µg ml^−1^ for tigecycline, 8–64 µg ml^−1^ for imipenem, and 0.5–4 µg ml^−1^ for clarithromycin (Table 1). Stocks of each isolate were preserved at −80 °C in skim milk and were thawed for each assay.

**Table 1:** Minimum inhibitory concentrations (MICs) of the *M. abscessus* clinical isolates studied

Isolate	MICs (µg ml^−1^)			
	AMK	TGC	IPM	CLR
*M. abscessus* 1	8	0.125	64	0.5
*M. abscessus* 2	8	0.5	16	4
*M. abscessus* 3	16	0.25	8	1
*M. abscessus* 4	16	0.25	64	0.5
*M. abscessus* 5	8	0.25	8	2
*M. abscessus* 6	8	0.25	32	0.5
*M. abscessus* 7	4	0.5	8	0.5
*M. abscessus* MIC_90_	16	0.5	64	4

*AMK* amikacin, *TGC* tigecycline, *IPM* imipenem, *CLR* clarithromycin

### Antibiotics

The four antibiotics tested were purchased from Sigma-Aldrich (St. Louis, MO, USA). Amikacin and imipenem were dissolved in sterile distilled water. Clarithromycin was dissolved in dimethyl sulfoxide (DMSO) (0.002% final concentration) and sterile distilled water. All the antibiotics were sterilized by filtration and stored at −20 °C.

### Tigecycline preparation

Tigecycline was prepared as described by Jitkova et al. [10] due to its poor stability. It was dissolved in DMSO (1 mg ml^−1^) and saline solution containing ascorbic acid (3 mg ml^−1^) (Sigma-Aldrich) and pyruvate (60 mg ml^−1^) (Sigma-Aldrich) and adjusted to pH 7.0. With this formulation, tigecycline remained stable for up to 7 days when protected from light [10].

### Inoculum preparation

All of the isolates were grown in BD^TM^ Columbia Agar with 5% Sheep Blood plates (Becton Dickinson, Sparks, MD). Then, they were subcultured in Middlebrook 7H9 liquid medium (Becton Dickinson) supplemented with 10% oleic acid-albumin-dextrose-catalase (Comercial Bellés, Tarragona, Spain) and 0.25% Tween 80 (Merck, Darmstadt, Germany) to avoid bacilli clump formation. Finally, the *M. abscessus* subsp *abscessus* cultures were homogenized by agitation and adjusted to the desired concentration using a nephelometer (CrystalSpec™; Becton Dickinson).

### Minimum inhibitory concentrations

The MICs of each antibiotic were determined in 96-well plates (Smartech Biosciences, Barcelona, Spain). Briefly, 100 µl of Mueller Hinton broth were added to each well. Then, serial dilutions of the antibiotics ranging from 512 to 0.25 µg ml^−1^ were made. Finally, 100 µl of inoculum at a concentration of 0.5 McFarland (5 × 10^6^ CFU ml^−1^) were added. Positive control wells consisted of 100 µl of Mueller Hinton and 100 µl of inoculum (5 × 10^6^ CFU ml^−1^). Negative control wells were prepared by adding 200 µl of Mueller Hinton. Plates were incubated at 30 °C for 3 days and for up to 14 days in the case of clarithromycin. After incubation, visual reading was performed. The MIC was interpreted as the lowest antibiotic concentration preventing growth. All the experiments were performed in duplicate.

### Time-kill assays

A previously described protocol was adapted for the present study [11]. Briefly, the time-kill assays were performed by dispensing 600 µl of the corresponding antibiotic concentration (amikacin, tigecycline, imipenem, or clarithromycin) and 500 µl of inoculum (final concentration of 5 × 10^5^ CFU ml^−1^) into tubes with 7.8 ml of Mueller Hinton broth. For the three-drug combinations (amikacin/tigecycline/imipenem and amikacin/tigecycline/clarithromycin), 200 µl of each of the three antibiotics were dispensed into the tubes. Amikacin and imipenem were tested at 2× MIC and tigecycline and clarithromycin at 4× MIC. Control growth tubes containing 500 µl of sterile distilled water instead of antibiotic were also included. All of the tubes were incubated at 37 °C in a 5% CO_2_ atmosphere for 6 days. Time points were established at days 0, 1, 3, and 6. At the defined time points, a volume of 500 µl was removed from each liquid culture. The number of viable mycobacteria in each culture was determined by plating 10-fold serial dilutions on Middlebrook 7H11 medium (Becton Dickinson). The 7H11 agar plates were incubated at 37 °C in a 5% CO_2_ atmosphere for 3 days, after which colony-forming units were counted. All the experiments were performed in duplicate.

### Data analysis of the time-kill assays

The means of the log_10_ CFU ml^−1^ values were plotted against time for each isolate. The results were interpreted by the effect of the combinations compared with the most active antibiotic tested individually. Synergy was considered when the activity of the combinations was 2 log_10_ higher than the most active antibiotic alone. Antagonism was determined when the activity of the combinations was 2 log_10_ lower compared to the most active antibiotic alone. Finally, a difference of less than 2 log_10_ lower or higher was considered indifferent.

### Statistical analysis

Significant differences between the two combinations (amikacin/tigecycline/imipenem or amikacin/tigecycline/clarithromycin) were analyzed using the Wilcoxon Mann–Whitney test. Results showing *P* ≤ 0.05 were considered as statistically significant. Calculations were performed using STATA 13.0 software (Stata Corporation, College Station, TX, USA).

## Results

Four different antibiotics, amikacin, tigecycline, imipenem, and clarithromycin, alone and in combination (amikacin/tigecycline/imipenem and amikacin/tigecycline/clarithromycin) were tested against clinical isolates of *M. abscessus* subsp. *abscessus* using the time-kill assay. The results observed during the 6-day period are summarized in Table 2.

**Table 2:** Mean colony-forming values (log_10_ CFU ml^−1^) and reduction in growth compared with the initial inoculum (∆) at the defined time points of the time-kill assay

Antibiotic or antibiotic combination	Day 0 (log_10_ CFU ml^−1^)	Day 1 (log_10_ CFU ml^−1^)	∆Day 1	Day 3 (log_10_ CFU ml^−1^)	∆Day 3	Day 6 (log_10_ CFU ml^−1^)	∆Day 6
GC	6.85 ± 0.09	7.35 ± 0.42	0.5	8.18 ± 0.39	1.33	8.80 ± 0.65	1.95
AMK	6.85 ± 0.10	6.37 ± 0.38	−0.48	5.62 ± 0.49	−1.23	4.84 ± 0.92	−2.01
TGC	6.85 ± 0.11	6.73 ± 0.43	−0.12	6.50 ± 0.33	−0.35	6.39 ± 0.31	−0.46
IPM	6.85 ± 0.12	6.12 ± 0.64	−0.73	5.47 ± 0.94	−1.38	5.37 ± 0.35	−1.48
CLR	6.85 ± 0.13	6.58 ± 0.42	−0.27	6.02 ± 0.40	−0.83	5.58 ± 0.60	−1.27
AMK/TGC/IPM	6.85 ± 0.14	6.10 ± 0.75	−0.75	5.42 ± 0.60	−1.43	4.27 ± 0.67	−2.58
AMK/TGC/CLR	6.85 ± 0.15	6.20 ± 0.49	−0.65	5.46 ± 0.70	−1.39	3.83 ± 0.90	−3.02

*CFU* colony-forming unit, *GC* growth control, *AMK* amikacin, *TGC* tigecycline, *IPM* imipenem, *CLR* clarithromycin

In the present study at day 6, amikacin and tigecycline showed a mean 2.01 and 0.46 log_10_ CFU ml^−1^ decrease, respectively, compared with the initial inoculum (5.70 log_10_ CFU ml^−1^). Imipenem and clarithromycin displayed a mean 1.48 and 1.27 log_10_ CFU ml^−1^ decrease, respectively. The combination of amikacin/tigecycline/imipenem showed a mean decrease of 2.58 log_10_ CFU ml^−1^. Finally, the combination of amikacin/tigecycline/clarithromycin displayed a mean decrease of 3.02 log_10_ CFU ml^−1^. Although being less pronounced, these decreases were also observed at day 3.

No significant differences were observed among the two replicates of each isolate. The most active individual antibiotic was amikacin followed by imipenem and clarithromycin. Both combinations were indifferent (neither synergistic nor antagonistic) showing more activity than the antibiotics tested individually. Moreover, no significant differences (*P* ≥ 0.05) were observed between the activities of the two three-drug combinations.

## Discussion

In this study, the effects of amikacin, tigecycline, imipenem, and clarithromycin individually and in two three-drug combinations were investigated as these antibiotics are usually administered during the treatment of lung infections caused by *M. abscessus*. To our knowledge, there are few studies on the effect of these antibiotics against *M. abscessus* clinical isolates using the time-kill assay [12, 13]. This method is adequate for studying the activity of antibiotics and determining their pharmacodynamics [14]. Furthermore, the time-kill assay has been largely used in clinical microbiology laboratories and has demonstrated to be reliable and consistent in the study of different microorganisms, including NTM [11, 15–18]. The antibiotic susceptibility testing for *M. abscessus* is read at day 3 in microbiological practice. However, in this study the time points were established at days 3 and 6 in order to study the accumulated activity of the antibiotics alone and of the three-drug combinations. Regarding the antibiotics, it is well-known that tigecycline has poor stability. In this study, it was prepared as described by Jitkova et al. and remained stable for up to 7 days [10]. This novel formulation allows the testing of tigecycline, a drug frequently used in the treatment of *M. abscessus* lung infections given that it is more effective than doxycycline and other tetracyclines [8]. Concerning imipenem, it shows poor stability in formulations parenterally administered to patients. This poor stability has been attributed to changes in pH and in the concentrations of sodium bisulfite and l-cysteine [19]. Nonetheless, our in vitro results showed activity of all the antibiotics and antibiotic combinations at both days 3 and 6.

Currently, the majority of patients with lung infections by *M. abscessus* receive more than two antibiotics in combination during the course of treatment. However, there is no standard combination and most of the treatments are based on empirical experience. In addition, most studies only investigate the activity of antibiotics in two-drug combinations. Some of them include few of the antibiotics analyzed in the present study, but none includes three-drug combinations [12, 15, 16].

In this study we used isolates of *M. abscessus* subsp. *abscessus*, the most isolated subspecies in our area. From a strain collection we selected seven clinical isolates susceptible to macrolides, with the aim to compare the two three-drug combinations. The results show that both combinations had good activity and did not show statistically significant differences, although the reduction in log_10_ CFU ml^−1^ with the combination including imipenem was lower than of that including clarithromycin. For this reason and due to the IV administration of imipenem, the combination of amikacin/tigecycline/imipenem could not be recommended as an alternative in the treatment of *M. abscessus* subsp. *abscessus* lung infections caused by clarithromycin susceptible isolates. However, in the light of the results obtained, this combination could be considered against clarithromycin resistant isolates. Future studies are required to confirm this hypothesis. This is especially important, since at least 14 months of treatment are necessary and outcomes remain very poor, with success rates of only 30–50% [20].

Furthermore, systems of antibiotic administration that have already proven to be effective against NTM lung infections, such as inhaled liposomal antibiotics and other nanoparticle-based antibiotic delivery systems, should be further developed. These forms of administration are currently used for amikacin [21]. In addition, imipenem physicochemical properties also allow its administration using these new delivery systems [22].

In conclusion, both three-drug combinations, amikacin/tigecycline/imipenem, and amikacin/tigecycline/clarithromycin, showed similar in vitro activity against *M. abscessus* subsp. *abscessus* clinical isolates as well as no antagonistic activity. The combination including imipenem is not a reliable alternative against *M. abscessus* subsp. *abscessus* lung infections caused by isolates susceptible to clarithromycin. However, this combination should be further studied against clarithromycin resistant isolates.
